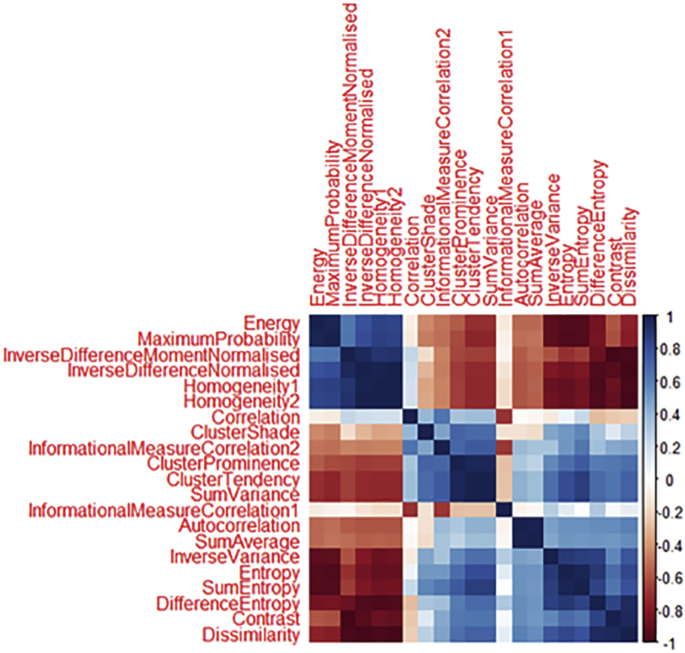# Radiomic features of cervical cancer on T2-and diffusion-weighted MRI: Prognostic value in low-volume tumors suitable for trachelectomy

**DOI:** 10.1016/j.ygyno.2019.10.010

**Published:** 2020-01

**Authors:** Benjamin W. Wormald, Simon J. Doran, Thomas EJ. Ind, James D'Arcy, James Petts, Nandita M. deSouza

**Affiliations:** a MRI Unit, Division of Radiotherapy and Imaging, The Institute of Cancer Research and the Royal Marsden NHS Foundation Trust, Sutton, UK; b Department of Gynaecological Oncology, The Royal Marsden NHS Foundation Trust, London, UK; c St George's University of London, Tooting, London, UK

**Keywords:** Radiomics, MRI, Cervical cancer, Recurrence, Trachelectomy, ADC, Apparent diffusion co-efficient, ROI, Region of interest, AUC, Area under curve, DW, Diffusion weighted, GLCM, Grey Level co-occurrence matrix, ROC, Receiver operating curve, LLETZ, Large loop excision of transformation zone, LVSI, Lymphovascular space invasion, SNR, Signal to noise ratio

## Abstract

**Background:**

Textural features extracted from MRI potentially provide prognostic information additional to volume for influencing surgical management of cervical cancer.

**Purpose:**

To identify textural features that differ between cervical tumors above and below the volume threshold of eligibility for trachelectomy and determine their value in predicting recurrence in patients with low-volume tumors.

**Methods:**

Of 378 patients with Stage1–2 cervical cancer imaged prospectively (3T, endovaginal coil), 125 had well-defined, histologically-confirmed squamous or adenocarcinomas with >100 voxels (>0.07 cm^3^) suitable for radiomic analysis. Regions-of-interest outlined the whole tumor on T2-W images and apparent diffusion coefficient (ADC) maps. Textural features based on grey-level co-occurrence matrices were compared (Mann-Whitney test with Bonferroni correction) between tumors greater (n = 46) or less (n = 79) than 4.19 cm^3^. Clustering eliminated correlated variables. Significantly different features were used to predict recurrence (regression modelling) in surgically-treated patients with low-volume tumors and compared with a model using clinico-pathological features.

**Results:**

Textural features (Dissimilarity, Energy, ClusterProminence, ClusterShade, InverseVariance, Autocorrelation) in 6 of 10 clusters from T2-W and ADC data differed between high-volume (mean ± SD 15.3 ± 11.7 cm^3^) and low-volume (mean ± SD 1.3 ± 1.2 cm^3^) tumors. (p < 0.02). In low-volume tumors, predicting recurrence was indicated by: Dissimilarity, Energy (ADC-radiomics, AUC = 0.864); Dissimilarity, ClusterProminence, InverseVariance (T2-W-radiomics, AUC = 0.808); Volume, Depth of Invasion, LymphoVascular Space Invasion (clinico-pathological features, AUC = 0.794). Combining ADC-radiomic (but not T2-radiomic) and clinico-pathological features improved prediction of recurrence compared to the clinico-pathological model (AUC = 0.916, p = 0.006). Findings were supported by bootstrap re-sampling (n = 1000).

**Conclusion:**

Textural features from ADC maps and T2-W images differ between high- and low-volume tumors and potentially predict recurrence in low-volume tumors.

## Highlights

• Texture features differed significantly between high-compared to low-volume cervical tumors (p < 0.02).
• In low-volume tumors predicting recurrence from ADC-radiomics was superior to T2W-radiomics or clinico-pathologic features.
• Combining ADC-radiomics and clinico-pathologic features together improved recurrence prediction further.

## 1 Introduction

Stage 1 cervical cancer is primarily treated with hysterectomy, although less radical surgical options (cone biopsy, trachelectomy) are considered where fertility preservation is desirable [[1], [2], [3], [4]]. Decisions regarding the type and extent of surgery and the subsequent need for adjuvant therapy depend on tumor resectability and the risk of recurrence. Biomarkers that predict recurrence, therefore, are of paramount importance for selecting the most appropriate treatment options. In tumors >2 cm in longest dimension, pre-operative tumor volume is a powerful adverse prognostic factor associated with reduced overall survival [5,6]. Other prognostic factors, such as tumor type, grade, lymphovascular space invasion (LVSI) and depth of stromal invasion are derived from a biopsy [[7], [8], [9], [10]], and therefore may not represent the tumor in its entirety. Prognostic biomarkers derived from imaging would be more representative of the whole tumor and would enable selection of the optimal surgical management at the outset in Stage 1 disease.

Magnetic Resonance imaging is routinely used to detect and stage cervical cancer, where T2-W and diffusion-weighted (DW) imaging form the mainstay of diagnostic sequences [11,12]. Derivation of an apparent diffusion coefficient (ADC) from the DW images [13] and analysis of first order histogram distribution of ADC values has been shown to predict histological subtype [14,15], staging [16], parametrial invasion [17], LVSI [18] the response to chemo-radiotherapy [19] and to aid surgical decision-making [20]. However, these first-order statistical quantitative imaging data remain limited in their prediction of likely recurrence [21]. It is possible to refine image analysis and convert the T2-W [22] and DW [23] imaging data into a high-dimensional feature space using algorithms to extract a more extensive set of statistical features within the data. This type of analysis, referred to as “radiomics”, requires that the data have a high signal-to-noise ratio to reduce error in the analysis from image noise; this is achievable in cervical cancer using an endovaginal MRI technique [24]. The purpose of this study was to identify radiomic features of cervical cancers on endovaginal MRI that differed between tumors below and above the volume threshold of eligibility for trachelectomy (less or greater than 4.19 cm^3^, equivalent to a 2 cm diameter spherical tumor volume) and to determine their value in predicting recurrence in patients in the low-volume tumor group.

## 2 Methods

### 2.1 Study design

This single-institution, prospective, pilot cohort study included patients with histologically confirmed cervical cancer, presumed Stage 1 or 2 (FIGO 2009 [25], referred for endovaginal MRI between March 2011 and October 2018 and potentially suitable for surgical management (trachelectomy or hysterectomy). This was part of an on-going institutional review board (IRB) approved research study documenting imaging features of cervical cancer indicative of poor outcome (NCT01937533). All patients gave their written consent for use of their data. All patients were treated with curative intent with either surgery or chemoradiation following MRI and staging investigations. Surgical options included cold-knife cone, trachelectomy or hysterectomy depending on their suitability for fertility preservation and their desire for continued fertility. A pelvic lymphadenectomy was performed in all cases.

Clinico-pathological metrics recorded in each case were tumor volume, type, grade, LVSI, parametrial invasion, Depth of Invasion and lymph node metastasis. Patients were followed up for median of 35 months (3-92). Median time to recurrence was 7 months (3-62 months).

### 2.2 Study participant selection

378 consecutive patients were imaged over the defined study period. In 98 cases, tumor was not identified on MRI while in 127 cases tumor was poorly identified and volume was <0.07 cm^3^, (62 of these had negative histology). Of the remaining 153 patients, 10 had non-cervical origin tumors on histology, 12 had histology other than squamous or adenocarcinoma (clear cell or neuroendocrine histology), 2 had metastatic disease, in 3 the whole tumor was not within the imaged field-of-view, and 1 did not have a diffusion-weighted images (Supplementary data, Fig. S1). These 28 exclusions resulted in 125 patients with histologically confirmed residual squamous-or adeno carcinomas that could be defined on MRI and were therefore eligible for analysis. No patients had to be excluded on the grounds of image artefact degrading the data. In patients who underwent primary surgery, the post-operative histological diagnosis was taken as the gold-standard. In those who received chemoradiation therapy, their pre-treatment histological diagnosis was taken as the gold-standard. In assessing lymph node status, surgical pathology was the reference gold-standard in those undergoing surgery, and imaging (MRI or PET-CT) was the reference gold-standard in those treated with chemoradiation.

### 2.3 MRI protocol

All scans were performed on a 3.0 T Philips Achieva (Best, The Netherlands) with a dedicated in-house developed 37 mm ring-design solenoidal receiver coil that has been previously described [[20], [21], [24]]. Cervical position was determined at vaginal examination, after which the coil was inserted and placed around the cervix. Image distortion from susceptibility artefacts were reduced by aspiration of vaginal air via a 4 mm diameter tube (Ryles; Pennine Healthcare, London, England). The administration of Hyoscine butyl bromide (Buscopan) 20 mg IM  decreased artefacts from bowel peristalsis.

T2-W images were obtained in three planes orthogonal to the cervix: TR/TE 2750/80 ms (coronal and axial) and 2500/80 ms (sagittal); field of view (FOV) 100 mm × 100 mm; acquired voxel size 0.42 × 0.42 × 2 mm; reconstructed voxel size 0.35 × 0.35 × 2 mm; slice thickness 2 mm; slice gap 0.1 mm; 24 coronal and 22 sagittal slices; number of signal averages (NSA) 2. Additionally, matched Zonal Oblique Multislice (ZOOM) diffusion-weighted images (DWI) were acquired: TR/TE 6500/54 ms; b-values 0, 100, 300, 500, 800 s/mm^2^; FOV 100 × 100 mm; acquired voxel size 1.25 × 1.25 × 2 mm; reconstructed voxel size 0.45 × 0.45 × 2 mm; slice thickness 2 mm, slice gap 0.1 mm; 24 slices, NSA 1. ADC maps were automatically generated by the scanner software. These were compared with T2-W images to identify the presence and extent of a tumor within the cervix. Mass-lesions disrupting the normal cervical epithelial architecture that were intermediate signal-intensity on T2-W images with corresponding restriction on the ADC maps were recognized as tumor.

### 2.4 MRI analysis: extraction of texture features

**Fig. 1:**
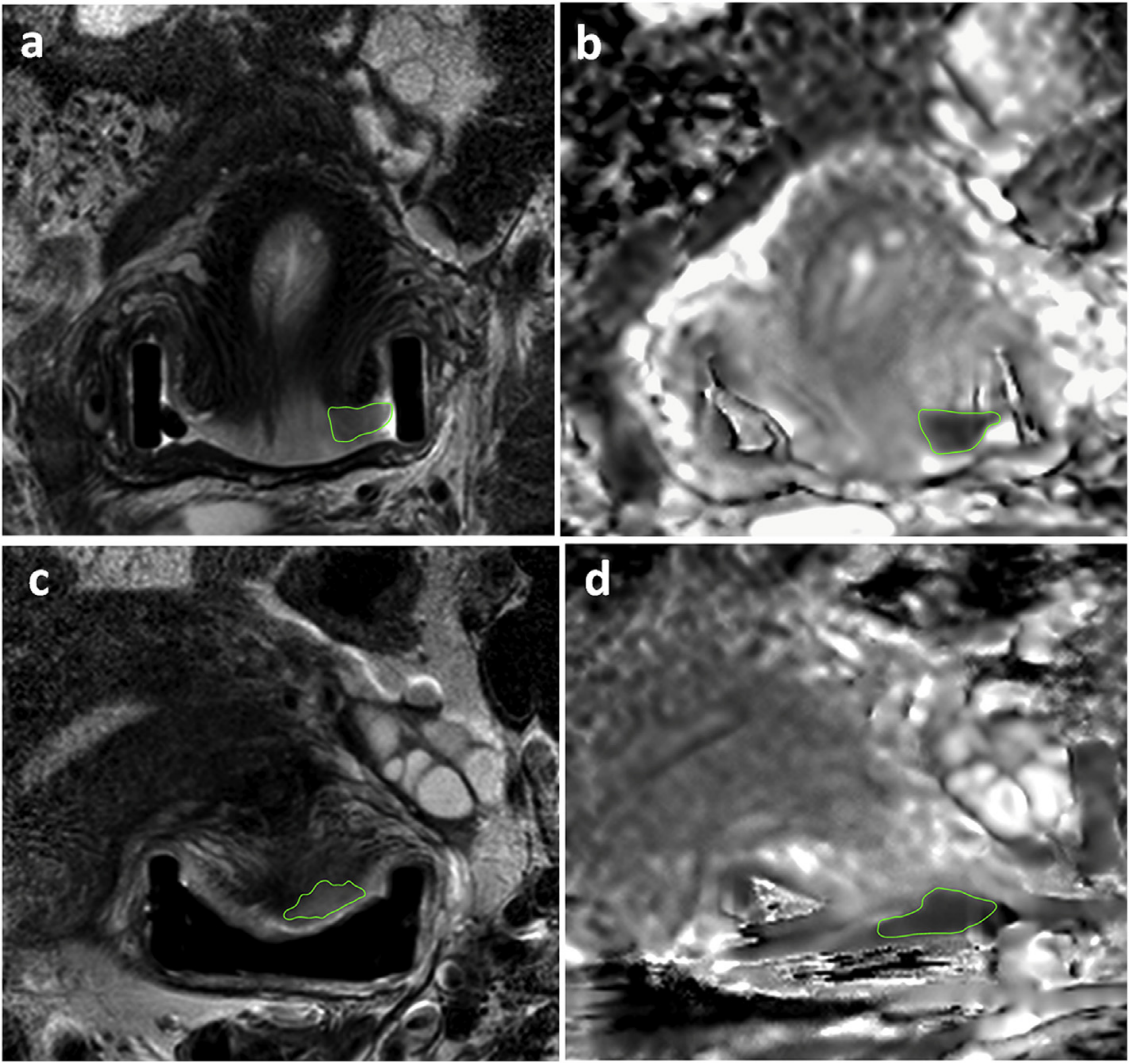
T2-W (a) and ADC map (b) in a 33- year old patient with a 0.8 cm^3^ volume tumor that had high dissimilarity (0.808). Regions-of-interest delineate the tumor. The intermediate signal-intensity tumor on the T2-W imaging is restricted in diffusion on the ADC maps. Tumor was confined to the cervix, and the patient remains disease-free following trachelectomy. T2-W (c) and ADC map (d) in a 26 year old patient with a 0.9 cm^3^ volume tumor that had low dissimilarity (0.489). The intermediate signal-intensity tumor on the T2-W imaging (c) is restricted in diffusion on the ADC maps (d). Regions-of-interest delineate the tumor. Tumor was confined to the cervix, but despite negative nodes on surgical histology, the patient recurred centrally after 9 months.

Scans were anonymised (DicomBrowser, Neuroinformatics Research Group, Washington University, St Louis, MO) and transferred to an XNAT [26,27] image repository. Images were imported into OsiriX (Pixmeo SARL, Bernex, Switzerland) and 2D regions-of-interest (ROI) were drawn by a radiologist, (25 years’ experience) on the coronal T2-W and ADC maps on every slice demonstrating tumor (Fig. 1). 2D ROI contours were aggregated using a custom Python script, integrated into OsiriX via pyOsirix [28] and exported as a single 3D volume (VOI) in DICOM RT-STRUCT format, which was then uploaded to XNAT. Custom in-house software (MATLAB, MathWorks, Natick, MA) was used to extract Grey Level Co-occurrence Matrix (GLCM) features (Haralick texture analysis [29,30]) from the both the T2-W images and ADC maps.

### 2.5 Statistical analysis

Statistical analysis was performed with *R* (R Core Team (2019), Vienna, Austria. http://www.R-project.org/). Correlations between features indicated 10 distinct feature clusters by creating a dissimilarity measure from a distance matrix (Supplementary data, Fig. S2). Several of the texture features were very highly correlated (r = 0.97–1) and were successfully clustered. The feature with the greatest dynamic range from each cluster was selected for investigation (Table S1): these were Dissimilarity, Contrast, Energy, Entropy, ClusterProminence, ClusterShade, InverseVariance, Correlation, Autocorrelation and InformationalMeasureCorrelation2. Contrast and Entropy, although not clustered with Dissimilarity and Energy respectively, were highly correlated (R > 0.9), and were removed.

A Shapiro-Wilk test revealed that features did not have a normal distribution so non-parametric tests were employed. A Mann-Whitney (*U*) test with Bonferroni correction was applied to assess the differences in texture features between tumors greater than or less than 4.19 cm^3^ on T2-W imaging (volume threshold of eligibility for trachelectomy, designated as high-volume and low-volume tumors). A p-value <0.05 was taken to be significant. Stepwise logistic regression was used to determine which combination of features from each category (ADC-radiomics, T2-W-radiomics and clinico-pathological metrics) were indicative of recurrence. This was done in 2 scenarios i) in all patients with low-volume tumors using adjuvant therapy as a feature in the model; ii) in only those patients who did not receive adjuvant therapy. The logistic regression coefficients were used to combine the features identified from each scenario to generate Receiver operating characteristic (ROC) curves for ADC-radiomic features and for T2-W radiomic features predicting recurrence in low-volume tumors. These were compared with the ROC curve of the clinico-pathological features identified in both scenarios using the Akaike information criteria (AIC). Further improvements in predicting recurrence were investigated by combining the features identified in the ADC-radiomic and T2-W radiomic models with the clinico-pathological features and evaluated with a Chi-square test. A bootstrap resampling (n = 1000) procedure was performed to obtain estimates of optimism in the regression models to provide a bias-corrected AUC value through a Somers’ D rank correlation metric whereby AUC = (1 + Somers D)/2. The rms: Regression Modelling Strategies R package, version 5.1–0 was used.

## 3 Results

### 3.1 Patient demographics and clinical characteristics

Eligible patients were aged between 24-89 years (mean 38.4 years) at primary treatment. Initial diagnosis was made with biopsy in 77 patients and large loop excision of the transformation zone (LLETZ) in 48 patients. Biopsies confirming the presence of cancer were not large or deep enough to confirm tumor grade in 1 case or LVSI in 7.

Of 125 patients, 79 were low-volume (range 0.26 – 4.17 cm^3^, mean 1.3 ± 1.2 cm^3^); 70 were treated surgically and 9 with chemoradiation. Forty-six were high-volume (range 4.2–56.1 cm^3^, mean 15.3 ± 11.7 cm^3^); 7 were treated surgically and 39 with chemoradiation. Of the 70 patients with low-volume tumors treated surgically, 2 patients did not have follow-up data, so that prediction of recurrence was modelled on 68 patients (Fig. S1). Patient and tumor characteristics in those with high- and low-volume tumors are detailed in Table 1.

**Table 1:** Patient characteristics for all tumors and for low- and high-volume tumor sub-groups (*1 treated with chemoradiotherapy).

	All tumors	High volume >4.19 cm^3^	Low volume <4.19 cm^3^
**Age, mean (range)**	38.4 (65.0)	43.0 (64.0)	35.6 (38.0)
**BMI, mean (range)**	25.7 (36.3)	26.2 (36.3)	25.4 (32.9)
**FIGO stage, n**			
1	74	69	5
2	51	10	41
**Histological subtype, % patients (n)**			
Squamous	61.6 (77)	78.3 (36)	51.9 (41)
Adenocarcinoma	38.4 (48)	21.7 (10)	48.1 (38)
**Grade % patients (n)**			
1 or 2	55.2 (69)	52.2 (24)	57.0 (45)
3	43.2 (54)	43.5 (20)	43.0 (34)
Unknown	1.6 (2)	4.3 (2)	0
**LVSI, % patients (n)**			
Positive	27.2 (34)	15.2 (7)	34.2 (27)
Negative	65.6 (82)	67.4 (31)	64.6 (51)
Unknown	7.2 (9)	17.4 (8)	1.2 (1)
**Depth of Invasion, mean (range)**	7.1 (20.4)	6.0 (19.0)	7.4 (20.4)
**Parametrial invasion % patients (n)**			
Positive	32.8 (41)	76.1 (35)	7.6 (6)
Negative	67.2 (84)	23.9 (11)	92.4 (73)
**Lymph node metastasis, % patients (n)**			
Positive	31.2 (39)	58.7 (27)	15.2 (12)
Negative	68.8 (86)	41.3 (19)	84.8 (67)
**Treatment, % patients (n)**			
Surgery	61.6 (77)	15.2 (7)	88.6 (70)
Chemoradiation	38.4 (48)	84.8 (39)	11.4 (9)
**Surgery, % patients (n)**			
Cold Knife Cone CKC	0	0	0
Trachelectomy	48.1 (37)	14.3 (1)	51.4 (36)
Hysterectomy	51.9 (40)	85.7 (6)	48.6 (34)
**Adjuvant treatment after surgery % patients (n)**			
Yes	23.4 (18)	28.6 (2)	22.9 (16)
**Recurrence, % patients (n)**			
Yes	16.0 (20)	26.1 (12)	10.1 (8)*
No	78.4 (98)	65.2 (30)	86.1 (68)
Unknown	5.6 (7)	8.7 (4)	3.8 (3)

Fifty-four of 68 patients in the low-volume group did not receive adjuvant therapy. Fourteen patients in the low-volume group received adjuvant therapy following surgery because of adverse features: 5 had unexpected lymph node metastases, 3 had unexpected extension of tumor to the parametrium, 1 had a 0.5 mm margin to the parametrium at surgical histology, 1 had spread to the vaginal cuff and 4 met 2 of the Sedlis criteria (LVSI) and deep stromal invasion). There were 7 recurrences overall: 5 in 54 patients who had not and 2 in14 in patients who had received adjuvant therapy.

### 3.2 Differences in texture features based on tumor volume and clinico-pathological metrics

Number of voxels in the T2-W images ranged from 17441-209892 in the high-volume tumors (median 38597) to 107-17324 in the low-volume tumors (median 2750). Number of voxels in the ADC maps ranged from 10497-140650 in the high-volume tumors (median 26812) to 75-13294 in the low-volume tumors (median 1927).

From heat-maps of correlated texture features (Supplementary data, Fig. S2), ten texture feature clusters were identified (Supplementary data, Table S1). After Bonferroni correction, 6 texture features on both ADC maps and T2-W images (Table 2) remained significantly different between the high- and low-volume tumors, namely Dissimilarity, Energy, ClusterProminence, InverseVariance and Autocorrelation. An additional feature on T2-W imaging (Correlation) differed between groups (Table 2).

**Table 2:** Texture features derived from ADC maps and T2-W images showing differences between low- and high-volume tumors.

Texture Feature		Median low volume N = 79	IQR low volume N = 79	Median high volume N = 46	IQR high volume N = 46	Adjusted p-value
Dissimilarity	ADC	0.64	0.28	0.35	0.17	1.22E-11
	T2W	0.49	0.32	0.25	0.12	4.31E-14
Energy	ADC	0.15	0.11	0.30	0.21	3.76E-09
	T2W	0.20	0.13	0.34	0.2	7.55E-10
InverseVariance	ADC	0.41	0.08	0.29	0.13	2.84E-11
	T2W	0.38	0.12	0.23	0.10	9.61E-13
ClusterProminence	ADC	29.33	40.77	10.52	10.11	5.95E-08
	T2W	22.66	24.14	8.03	6.50	1.49E-09
ClusterShade	ADC	2.82	3.62	1.26	1.42	3.84E-03
	T2W	2.29	2.28	1.18	1.30	0.02
Autocorrelation	ADC	11.41	5.68	9.13	4.64	0.02
	T2W	11.65	8.69	6.08	3.64	8.22E-09
InformationalMeasure Correlation2	ADC	0.63	0.21	0.54	0.07	0.08
	T2W	0.67	0.16	0.68	0.22	1
Correlation	ADC	0.44	0.18	0.47	0.07	0.89
	T2W	0.55	0.23	0.62	0.26	0.03

In low-volume tumors, Dissimilarity and Energy differed in patients without and with LVSI. (Supplementary data, Table S2). However, none of the Haralick features from ADC maps or T2-W images differed between adeno- and squamous cancers, low and high-grade tumors, or those with negative vs. positive lymph node status.

### 3.3 Clinico-pathological features as predictors of recurrence

AUCs and 95% CI for individual clinico-pathological features for predicting recurrence in all low-volume tumors (n = 68) regardless of adjuvant therapy were: (tumor type (0.548 [0.340–0.756]), grade (0.501 [0.294–0.709]), LVSI (0.537 [0.347–0.728]), Depth of Invasion (0.553 [0.291–0.814]), lymph node metastasis (0.530 [0.386–0.675]) and T2-W tumor volume (0.691 [0.448–0.934]). Adjuvant treatment had an AUC of 0.544 [0.357–0.732]. When patients receiving adjuvant therapy were excluded (n = 54), the AUCs were: tumor type (0.555 [0.305–0.805]), grade (0.504 [0.254–0.754]), LVSI (0.633 [0.570–0.695]), Depth of Invasion (0.510 [0.214–0.807]), lymph node metastasis (0.510 [0.490–0.530]) and T2-W tumor volume (0.629 [0.327–0.931]). Regression modelling which included adjuvant therapy as a confounding feature indicated that volume and Depth of Invasion were indicative of recurrence (AUC = 0.766 CI 0.562–0.970), but that when patients who received adjuvant therapy were excluded, LVSI alone was predictive of recurrence (AUC = 0.633 95% CI 0.570–0.695).

Combining T2-W volume, Depth of Invasion and LVSI predicted recurrence in all 68 low-volume tumors with an AUC 0.794 (95% CI 0.617- 0.971) and AIC of 45.684.

### 3.4 Texture features from ADC maps as predictors of recurrence

**Fig. 2:**
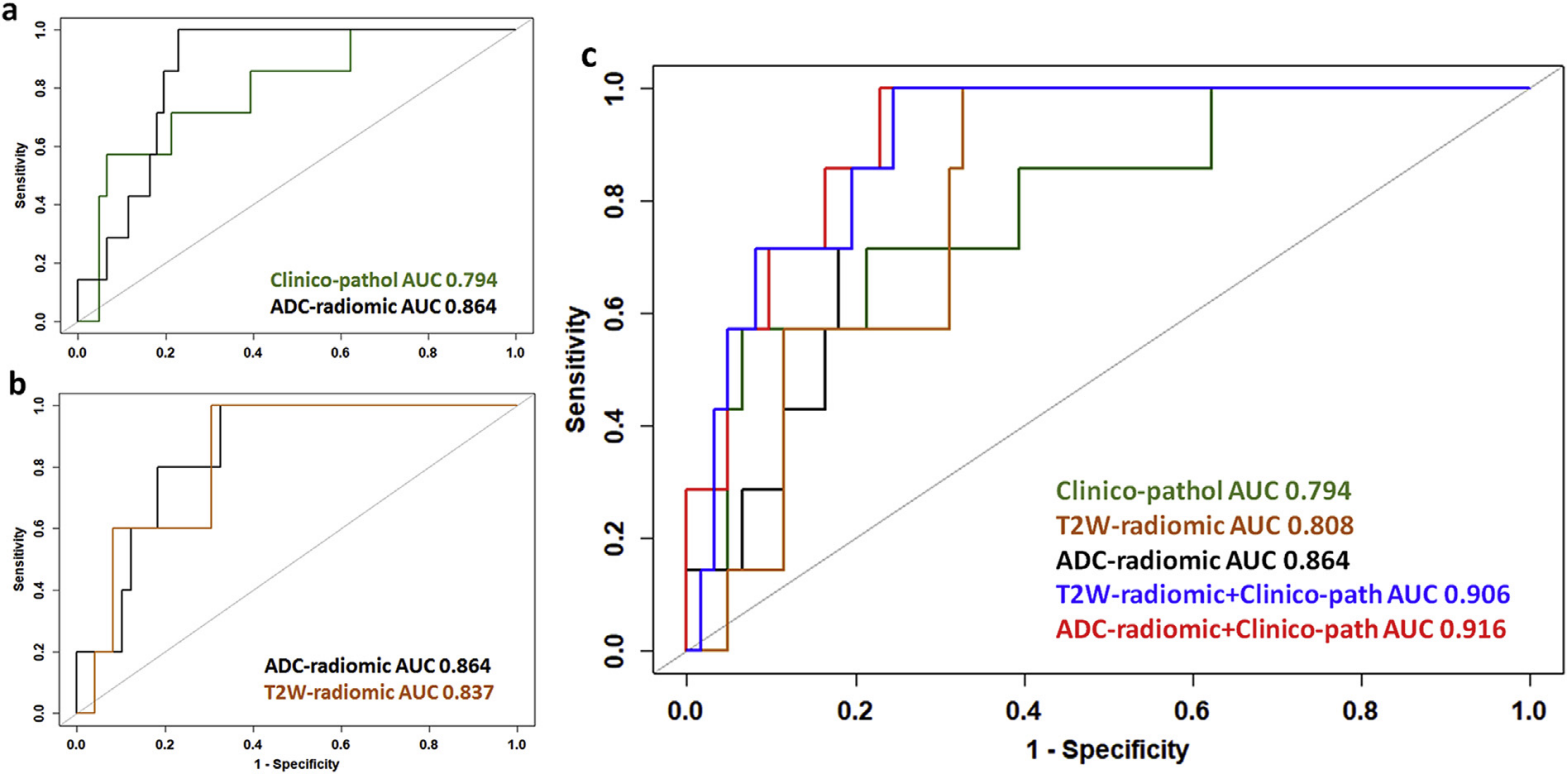
Receiver Operating Curves showing sensitivity and specificity for prediction of recurrence by texture and clinic-pathological features (a) in 68 patients with low-volume tumors where use of adjuvant therapy is included in the model; (b) in 54 patients who did not receive adjuvant therapy; and (c) in all 68 patients using features identified in both a and b (Dissimilarity, Energy for ADC-radiomics; Dissimilarity, ClusterProminence, InverseVariance for T2-W-radiomics; and Volume, Depth of Invasion, LymphoVascular Space Invasion for clinico-pathological features). In a, no combination of T2-W features was significantly superior to individual features. In b, of the clinico-pathological features, LVSI alone was predictive of recurrence, In c, the optimal prediction of recurrence is shown by a combination of ADC-radiomic and clinico-pathological features.

When considering all 68 patients with low-volume disease, the texture features Dissimilarity, Energy, InverseVariance, ClusterProminence, ClusterShade, Autocorrelation and volume derived from ADC maps had an AUC of 0.775, 0.635, 0.674, 0.646, 0.508, 0.665 and 0.672, respectively for predicting recurrence. (Fig. 2, Table 3). A regression model indicated that when combined, Dissimilarity and Energy were contributory to prediction of recurrence (AUC = 0.864, 95% CI = 0.772–0.956, AIC 41.044). However, when patients who had adjuvant therapy were excluded, only Dissimilarity was predictive of recurrence (AUC = 0.853, 95% CI = 0.725–0.981).

**Table 3:** Texture features derived from ADC maps and T2-W images in 68 low-volume tumors for prediction of recurrence.

Texture feature	From	Auc (ci)	Threshold	Sensitivity	Specificity
Dissimilarity	ADC map	0.775 (0.646–0.904)	0.635	100	61
	T2-W image	0.609 (0.334–0.883)	0.318	43	89
Energy	ADC map	0.635 (0.432–0.838)	0.178	71	61
	T2-W image	0.604 (0.373–0.835)	0.235	71	67
ClusterProminence	ADC map	0.646 (0.425–0.868)	53.789	100	33
	T2-W image	0.607 (0.364–0.849)	12.113	43	85
InverseVariance	ADC map	0.674 (0.496–0.853)	0.443	100	38
	T2-W image	0.665 (0.444–0.886)	0.349	71	66
Autocorrelation	ADC map	0.665 (0.497–0.833)	11.978	100	41
	T2-W image	0.628 (0.463–0.793)	8.921	100	38
Correlation	ADC map	-	-	-	-
	T2-W image	0.536 (0.326–0.746)	0.524	71	57
ClusterShade	ADC map	0.508 (0.292–0.724)	5.75	100	23
	T2-W image	0.511 (0.274–0.747)	3.474	86	26
InformationMeasureCorrelation2	ADC map	-	-	-	-
	T2-W image	-	-	-	-
Volume	ADC map	0.672 (0.426–0.919)	1292.136	71	64
	T2-W image	0.691 (0.448–0.936)	1248.191	71	64

Combining metrics predictive of recurrence from ADC-radiomic and clinico-pathological models (Dissimilarity and Energy with T2-W volume + Depth of Invasion + LVSI) significantly improved prediction of recurrence in all 68 low-volume tumors (AUC = 0.916, 95% CI 0.837–0.994, with 100% sensitivity, 77% specificity, p = 0.006, AIC = 39.638, Table 4) compared to the combined clinico-pathological model of T2-W volume + Depth of Invasion + LVSI.

**Table 4:** Regression models in prediction of recurrence with bootstrap corrected AUC and Chi-Square test of model differences. The reduction in AIC when ADC-radiomic and clinico-pathological features are combined compared to clinico-pathological features alone is indicative of the improvement of the combined model.

	AUC	CI	Corrected AUC	AIC	Resid. Df	Resid. Dev	Df	Deviance	p Value*
Clinico-pathological	0.794	0.617–0.971	0.708	45.684	64	37.684	-	-	-
ADC-Radiomic	0.864	0.772–0.956	0.824	41.044	65	-	-	-	-
T2W-Radiomic	0.808	0.690–0.926	0.716	49.193	65	-	-	-	-
ADC-Radiomic + Clinico-pathological	0.916	0.837–0.994	0.840	39.638	63	27.638	2	10.046	0.006
T2W-Radiomic + Clinico-pathological	0.906	0.822–0.991	0.822	45.128	61	31.128	3	6.556	0.086

*p-value of nested model compared to clinico-pathological model.

Examples of tumors with high Dissimilarity, and low Energy vs. low Dissimilarity and high Energy respectively are illustrated in Fig. 1.

### 3.5 Texture features from T2-W imaging as prognostic biomarkers

When considering patients with low-volume disease, the texture features Dissimilarity, Energy, InverseVariance, ClusterProminence, ClusterShade, Autocorrelation, Correlation and Volume derived from T2-W images individually had an area under the curve (AUC) of 0.609, 0.604,0.671, 0.607, 0.628, 0.536, 0.511 and 0.691 respectively for predicting recurrence (Table 3). When all low-volume tumors were considered, a regression model indicated that no combination of features improved prediction of recurrence. When patients who had adjuvant therapy were excluded, Dissimilarity, Clusterprominence and InverseVariance together were predictive of recurrence (AUC = 0.837, 95% CI = 0.698–0.976). These features applied to all 68 patients gave an AUC of 0.808 (95% CI = 0.690–0.926, AIC = 49.193).

Combining metrics predictive of recurrence from T2-W-radiomic and clinico-pathological models (Dissimilarity, ClusterProminence and InverseVariance with LVSI + Depth of Invasion + T2-W volume) did not significantly improve prediction of recurrence in low-volume tumors (AUC = 0.906, 95% CI 0.822–0.991, p = 0.09, AIC = 45.128, Table 4) compared to the combined clinico-pathological model of T2-W volume + Depth of Invasion + LVSI.

### 3.6 Validation of logistic regression models

Bias-corrected AUCs generated through a bootstrap resampling process showed reductions in AUC from 0.864 to 0.824 for the ADC-radiomic model (Dissimilarity and Energy), from 0.808 to 0.716 for the T2-W radiomic model (Dissimilarity, InverseVariance and ClusterProminence) and from 0.794 to 0.718 for clinico-pathological model (T2-W volume, Depth of Invasion and LVSI). The combined radiomic and clinico-pathological models were corrected from 0.916 to 0.84 (ADC-radiomic and clinico-pathological features) and from 0.906 to 0.822 (T2-W-radiomic and clinico-pathological features).

## 4 Discussion

Our data has identified the radiomic features from ADC maps and T2-W images that differ between high- and low-volume cervical tumors and shown that these features individually and in combination are useful for predicting recurrence in low-volume tumors. Patients in the high- and low**--**volume tumor groups were well matched by age, and although the low-volume tumors were by definition lower stage, there were more adenocarcinomas and LVSI in this group, both of which adversely affect outcome. Radiomic differences between high and low-volume tumors were largely similar for both the ADC and T2-W data although regression models identified different combinations of features as being contributory to prediction of recurrence in each case. Moreover, although radiomic features differed between tumors with and without LVSI, they did not differ between other histological parameters of poor prognosis (type, grade, Depth of Invasion, LN metastasis), indicating that they are likely to be independent.

This data highlights the potential of texture feature analysis for predicting recurrence with capability to influence the surgical management of patients with early stage, low-volume cervical cancer. It means that surgical management can be altered, or appropriate patient counselling provided at the outset because the use of adjuvant therapy can be anticipated. The utility of such information would be particularly valuable in a young patient population seeking to retain fertility and minimize therapy. For instance, to avoid the toxicity of lymphadenectomy followed by adjuvant chemoradiation, patients with “good” radiomic features may elect to have sentinel node biopsy prior to curative treatment (surgery or chemoradiation). Additionally, patients could be counselled as to the need for adjuvant therapy at the outset. In larger tumors, where volume is a strong predictive factor of recurrence [31] and survival [32], the utility of additional radiomic analyses in altering management remains to be established.

The greater tendency to decreased Dissimilarity in larger tumors, indicates that grey levels in adjacent pixels were similar in larger tumors. Energy, which is a measure of textural uniformity, and is highest when grey level distribution has either a constant or a periodic form, also was higher in larger tumors. A previous prospective study has confirmed the reproducibility of these features and their lack of dependence on regional ROI selection within the tumor [33], nevertheless we used whole tumor analysis in our study. A study by Hao et al. has shown that radiomic analysis of the tumor periphery is informative in differentiating those likely to recur from those that do not [34], but the tumor volume in their cohort was high and patients were treated with chemoradiation. Our data interrogates the differences in features between high-vs. low-volume tumors across the entire tumor volume and uses these features to recognize low-volume tumors with potentially poor prognosis. It confirms for the first time using radiomic analysis, that as small cervical tumors grow, they tend to become texturally less dissimilar and more homogenous. This may well reflect the transition from a morphology where tumor elements are interspersed with normal cervical glandular elements and stroma in smaller tumors to more homogenous sheets of malignant cells as tumors increase in size and de-differentiate. The T2W-radiomic features, however, were less good than the ADC-radiomic features for predicting recurrence. They did not offer significant improvements for prediction of recurrence when combined with clinico-pathological features as the model over-fitted the data. T2-W data also was affected by signal-intensity variations across the image, particularly in the presence of an endovaginal coil, which was not an issue with the quantified ADC from diffusion-weighted images.

Other retrospective studies have reported radiomic features derived from MRI and ^18^FDG-positron emission tomography (PET) scans of locally advanced cervical cancer treated with chemoradiotherapy. Radiomics features such as entropy from ADC maps and grey level non-uniformity from PET, respectively, have been shown to be independent predictors of recurrence and loco-regional control in these larger volume tumors with significantly higher prognostic power than usual clinical parameters [35].This supports our findings where these features are shown to differ between high- and low-volume tumors and to be predictive of recurrence in the low-volume tumor group.

A strength of this study was the derivation of the data using an endovaginal receiver coil, particularly in small volume tumors where it was possible to obtain a minimum of 100 voxels. This provided a substantial boost in SNR [24] and was invaluable for the assessment of the ADC data where external array imaging in the low-volume tumors would have limited the voxel numbers and precluded meaningful ADC feature analysis.

The application of adjuvant therapy as a confounding factor represented an analysis dilemma: removal of patients with low-volume tumors on MRI who went on to receive adjuvant chemotherapy would have biased the sample and made it unrepresentative of the final application. On the other hand, retaining these patients in the analysis, potentially weakened the model because patients with MRI radiomics features indicative of a recurrence after surgery will have that recurrence prevented by the adjuvant treatment. Our solution here was to perform both sets of analyses. As predicted, when the patients who received adjuvant therapy were removed, the AUC of the model increased, but at the cost of a smaller sample size.

Like many current studies in tumor radiomics, our work has several limitations. First it is a single site study with a relatively small sample size, albeit from a quaternary referral gynaecological oncology centre which sees and treats a high volume of patients. Second, the recurrence rate was low (∼10%) but is in keeping with expectations in this early stage, potentially curable disease. Even with a larger sample size, it would not have been possible to avoid such an imbalance between the recurrence and no-recurrence classes. Taken together, these factors lead to a model based on a small number of recurrences and the consequent risk of overfitting from the combined model, with a possibly over-optimistic value for the combined-model AUC. However, we show that for *single-feature* models *any one* of the ADC radiomic features Dissimilarity, Energy, InverseVariance, ClusterProminence, Autocorrelation or ADC volume performed better than the highest-scoring “clinico-pathological” features (T2-W volume and LVSI). Furthermore, when considering models based on just *two* features, the radiomic model (ADC Dissimilarity and Energy, AUC = 0.864) compared well with the clinical model (T2-W volume and Depth of invasion, AUC = 0.766). Third, patients were often diagnosed following a LLETZ biopsy which may remove a significant volume of disease, thus affecting the assessment at their staging MRI and confounding our results; this was the case in 1 patient in our study group. Nevertheless, in a clinical setting a LLETZ or cone biopsy is performed as part of the normal clinical pathway prior to MRI and imaging prior to a diagnostic LLETZ or cone biopsy is unlikely, making our results more applicable in a clinical workflow. In future, when determining the utility of radiomic features combined with other clinical and histologic assessments, use of MRI plus LLETZ volume is desirable. Finally, the current poor availability of endovaginal MRI limits radiomic assessments of low-volume tumors more widely. However, if further accumulation of cases confirms the predictive power of this model and that high SNR enables its implementation, this will provide a justification for more widespread use of this MRI technique at specialist centres offering trachelectomy. Alternatively, improvements of SNR in non-endovaginal MRI may be required.

In conclusion, in patients with low-volume tumors, ADC-radiomic texture analysis is potentially a useful predictor of tumor recurrence. This can substantially impact the treatment planning and counselling of patients with low-volume tumors seeking fertility preservation. The regression model derived from this data requires validation in a test set. It should then be possible to set thresholds for the relevant radiomic and clinical factors and to use these in a nomogram to predict the likelihood of recurrence in a clinical setting.

## Financial support

We gratefully acknowledge CRUK support to the Cancer Imaging Centre at ICR and RMH in association with MRC and Department of Health C1060/A10334, C1060/A16464 and NHS funding to the NIHR Biomedical Research Centre and the Clinical Research Facility in Imaging.

BW is funded by the CRUK Imaging Centre award C1060/A16464. SD and JD are funded by the CRUK National Translational Imaging Accelerator (NCITA, C7273/A28677) award.

## CRediT authorship contribution statement

**Benjamin W. Wormald:** Conceptualization, Methodology, Data curation, Formal analysis, Writing - original draft. **Simon J. Doran:** Methodology, Data curation, Formal analysis, Supervision. **Thomas EJ. Ind:** Data curation, Writing - review & editing. **James D'Arcy:** Software, Formal analysis. **James Petts:** Software, Formal analysis. **Nandita M. deSouza:** Conceptualization, Writing - review & editing, Supervision.

## Declaration of competing interest

All authors declare no potential conflicts of interest.

## Figures and Tables

**figs1:**