# Supplementary material for: Radiomic features of cervical cancer on T2-and diffusion-weighted MRI: Prognostic value in low-volume tumors suitable for trachelectomy
Source: Gynecol Oncol. 2020 Jan;156(1):107–14. doi: 10.1016/j.ygyno.2019.10.010 (PMC7001101; doi:10.1016/j.ygyno.2019.10.010)
Supplement: Multimedia component 1 [file mmc1.docx]

March 2011 – October 2018

**Endovaginal MRI scans n= 378**

MRI – no tumor present n= 98

Tumor poorly identified on MRI n= 127

Tumor present on MRI and post-operative specimen n= 153

Tumor out of field of view n=3

No DWI n=1

Histology non-cervical in origin, n= 10

Histology clear cell or neuroendocrine n= 12

Metastatic disease n= 2

**Low tumor volume < 4.19 cm^3^ n= 79**

**ELIGIBLE PATIENTS**

histologically confirmed squamous or adenocarcinoma of the cervix and whole tumor imaged with T2-W and DWI

**n= 125**

High tumour volume > 4.19 cm^3^ n= 46

Low volume – treated with surgery n= 70

Treated with chemo-radiation n = 9

Low volume – treated with surgery with follow up

**ANALYZED FOR PREDICTION OF RECURRENCE**

**n= 68**

Lost to follow-up n= 2

Tumor volume < 4.19 cm^3^

n= 79
