# Supplementary material for: Radiomic features of cervical cancer on T2-and diffusion-weighted MRI: Prognostic value in low-volume tumors suitable for trachelectomy
Source: Gynecol Oncol. 2020 Jan;156(1):107–14. doi: 10.1016/j.ygyno.2019.10.010 (PMC7001101; doi:10.1016/j.ygyno.2019.10.010)
Supplement: Multimedia component 2 [file mmc2.docx]

**Supplementary materials**

**Figure S1:** Flow chart showing criteria that determined final study cohort.

**Figure S2:** Heatmap of correlations between Haralick features

**Table S1:** Clusters of Haralick features that are significantly correlated and their dynamic ranges

| **Texture feature color coded by cluster** | **Dynamic range** |
| --- | --- |
| Aerts.Texture.InverseVariance | **14.18** |
| Aerts.Texture.Energy | **13.43** |
| Aerts.Texture.MaximumProbability | 7.62 |
| Aerts.Texture.Entropy | **4.87** |
| Aerts.Texture.SumEntropy | 4.24 |
| Aerts.Texture.Autocorrelation | **8.05** |
| Aerts.Texture.SumAverage | 2.95 |
| Aerts.Texture.Contrast | **106.39** |
| Aerts.Texture.InverseDifferenceMomentNormalised | 1.06 |
| Aerts.Texture.Dissimilarity | **39.41** |
| Aerts.Texture.DifferenceEntropy | 11.29 |
| Aerts.Texture.Homogeneity2 | 2.11 |
| Aerts.Texture.Homogeneity1 | 1.89 |
| Aerts.Texture.InverseDifferenceNormalised | 1.17 |
| Aerts.Texture.Correlation | **16.47** |
| Aerts.Texture.InformationalMeasureCorrelation1 | 0.13 |
| Aerts.Texture.ClusterShade | **-2.52** |
| Aerts.Texture.ClusterProminence | **177.40** |
| Aerts.Texture.ClusterTendency | 36.30 |
| Aerts.Texture.SumVariance | 36.30 |
| Aerts.Texture.InformationalMeasureCorrelation2 | **3.14** |

| Histologic feature | From | Dissimilarity | Energy | Cluster prominence | Inverse variance | Auto correlation | Correlation | Cluster  Shade | Informational  Correlation2 |
| --- | --- | --- | --- | --- | --- | --- | --- | --- | --- |
|  |  | ***Mann-Whitney U***  ***(p-value)*** | | | | | | | |
| Type | ADC map | 768 (0.152) | 414 (0.360) | 676 (1) | 796 (0.056) | 656 (1) | - | 692 (1) | 710 (0.848) |
|  | T2-W image | 708 (0.888) | 432 (0.600) | 699 (1) | 718 (0.688) | 744 (0.328) | 631 (1) | - | - |
| Grade | ADC map | 451 (1) | 679 (1) | 407 (0.400) | 446 (1) | 497 (1) | - | 394 (0.264) | 354 (0.064) |
|  | T2-W image | 504 (1) | 603 (1) | 547 (1) | 481 (1) | 460 (1) | 488 (1) | - | - |
| Parametrium | ADC map | 125 (1) | 87 (1) | 112 (1) | 114 (1) | 160 (0.512) | - | 77 (1) | 143 (1) |
|  | T2-W image | 135 (1) | 73 (1) | 160 (0.512) | 114 (1) | 150 (0.960) | 119 (1) | - | - |
| LVSI | ADC map | 756 (0.024) | 306 (0.032) | 680 (0.408) | 696 (0.248) | 699 (0.224) | - | 635 (1) | 699 (0.224) |
|  | T2-W image | 776 (0.008) | 324 (0.064) | 669 (0.568) | 775 (0.008) | 770 (0.016) | 450 (1) | - | - |
| Lymph node metastasis | ADC map | 277 (0.400) | 121 (1) | 238 (1) | 237 (1) | 200 (1) | - | 206 (1) | 198 (1) |
|  | T2-W image | 304 (0.088) | 84 (0.224) | 228 (1) | 302 (0.104) | 258 (0.976) | 65 (0.072) | - | - |

**Supplemental digital content 3.docx**

**Table S2:** Differences in texture features derived from ADC maps and T2-W images for low-volume tumors in patients with poor prognosis histologic features
